# Burnout and Maladjustment Among Employed Students

**DOI:** 10.3389/fpsyg.2022.825588

**Published:** 2022-04-22

**Authors:** Gabriela-Lăcrămioara Drăghici, Ana-Maria Cazan

**Affiliations:** Faculty of Psychology and Education Sciences, Transilvania University of Braşov, Braşov, Romania

**Keywords:** burnout, test anxiety, employed student, stress, academic maladjustment

## Abstract

Stress and burnout are present in every aspect of an individual’s life, and the growing number of employed students raises certain concerns about their engagement in academic tasks and finishing their studies. Our study aims to analyze the differences between student burnout in different contexts, work- and academic-related burnout, and examine the predictive role of burnout in academic maladjustment, including test anxiety as a mediator and occupational status as a moderator. The sample consisted of 151 students from different universities in Romania. Consistent with previous studies, the results showed that academic burnout is higher than work-related burnout. High levels of test anxiety explain high levels of academic burnout, which in turn explains low levels of academic adjustment. The results highlight the mediating role of anxiety in the relationship between academic burnout and academic maladjustment with occupational status as a moderator. Future research should focus on the type of students’ job, the mediating relationship between self-efficacy and academic burnout, and the relationship between burnout and personality traits.

## Introduction

Stress and burnout are present in every aspect of an individual’s life, and the growing number of employed students raises certain concerns about their engagement in academic tasks and finishing their studies. Students’ decision to work during their university years can be explained by the requirement of paying a high scholarship fee and meeting the living conditions. The inability to cope effectively with the challenges of the professional and academic life can have severe repercussions on a student’s mental health and well-being (Youssef and Luthans, 2007), with depression and suicide attempts being the two reactions to stress that are of concern to the society (Oswalt and Riddock, 2007). At the international level, student stress and burnout are seen as important health issues, with students being the population at risk of experiencing psychological distress and psychological disorders such as anxiety, depression, and panic disorder (Larcombe et al., 2016). Burnout among employed students is even higher, with studies showing that the stress caused by juggling the demands of work and academic life could potentially lead to burnout (Dyrbye et al., 2008) and depression (Njim et al., 2019), these negative effects being more prevalent among students who work for 20 or more hours per week (Larcombe et al., 2016). The potential stressors for a student are represented by the adaptation to the new status, accommodation with high academic requirements, financial and personal independence, and the establishment of a new social network (Hicks and Heastie, 2008). Burnout in students is related to both employment and academic domains; however, most studies on this topic consider employment as a major burnout factor, without exploring also additional individual characteristics that may impact burnout (Schramer et al., 2019). Higher levels of burnout can negatively impact academic adjustment. Academic adjustment refers to effectively regulating study behavior, being intrinsically motivated to learn, and satisfaction toward the chosen degree program and academic performance (van Rooij et al., 2018). College students face various psychological difficulties due to exposure to stressful situations; therefore, academic adjustment and burnout are strongly related. Numerous studies reveal the aspects linked to university adjustment, such as anxiety, depression, stress, vulnerability, anger, mood, and mental illness, indicative of negative adaptation (Clinciu, 2013).

Given the challenges faced by employed students, the main aim of this study was to analyze the differences between student burnout in different contexts, work- and academic-related burnout, focusing also on the predictive role of burnout in academic maladjustment, and including test anxiety as a mediator and occupational status as a moderator.

## Related Literature

### Academic- and Work-Related Burnout in Employed Students

Burnout is seen as a reaction to occupational stress that acts over a longer period of time (Gorter et al., 1999). Other authors introduced a multidimensional perspective to define the phenomenon of burnout, namely, emotional exhaustion, depersonalization, and reduction of personal achievements (Maslach and Leiter, 2017). Emotional exhaustion is defined as a state of emotional emptiness felt by an individual accompanied by the belief that his/her own resources are not sufficient to manage and adapt to the requirements of the environment (Maslach and Leiter, 2017). Depersonalization was later replaced by the term cynicism and manifests itself in the social sphere and describes the maladaptive attitudes and weak social skills of the individual, marked by a lack of interest in social contacts. Reducing personal achievements has been replaced by inefficiency, which is the individual’s belief that his or her cognitive and emotional efforts and resources are not sufficient to perform professional tasks (Maslach and Leiter, 2017).

Burnout manifests itself on a physical and psycho-behavioral level. Physical symptoms include increased heart rate, sleep disorders and changes in eating behavior, and decreased immunity. Psycho-behavioral symptoms include decreased ability to remember and concentrate, decreased decision-making, decreased interest and productivity, behavioral disorders (apathy, aggression), and mental disorders (depression, anxiety) (Misra and McKean, 2000). The consequences of burnout are found both emotionally and professionally. The decrease in self-esteem is the result of awareness of failure, inability to cope with the situation, and failure to meet their own expectations. People who show symptoms of emotional burnout are impatient, have an exaggerated critical spirit, are suspicious, and are convinced that those around them want to make their lives difficult. The long-term effects of the phenomenon are impaired health, low self-esteem, creating tensions in relationships with family, friends, and colleagues, decreased performance, and so on (Platon and Gorincioi, 2012).

Academic burnout is considered the result of long-term academic pressures and stress, energy consumption, and a gradual decrease of interest in academic activities due to work overload and extra activities or jobs. Even if burnout is a work-related term, researchers concluded that work can be replaced with “study” for students for a better description of their emotions and behaviors in pre-university and university environments (Lin and Huang, 2014). Academic burnout refers to “feeling exhausted because of study demands, having a cynical and detached attitude toward study, and feeling incompetent as a student” (Schaufeli et al., 2002, p. 465). Exhaustion and cynicism have been shown to be defining characteristics for college students with burnout (Neumann et al., 1990), adolescent students (Murberg and Bru, 2004), and manipulative burnout in the workplace (Schaufeli and Taris, 2005). Considered an erosion of student engagement (Fiorilli et al., 2017), student burnout also refers to feeling incompetent as a student, having the tendency to evaluate the educational context negatively (low personal accomplishment) (Schaufeli et al., 2002).

Burnout has many adverse consequences for students; however, the relationship between student burnout and academic adjustment is inconsistent (Madigan and Curran, 2021). While some studies reported negative relationships, students with a higher level of academic burnout obtain lower levels of academic performance (Fiorilli et al., 2017); other studies found no significant association between student burnout and future academic performance (Salanova et al., 2010). However, most of the studies have identified a negative association between burnout and academic achievement (Brougham et al., 2009), this relationship being moderated instead by a number of factors such as the coping strategies, the stage of education (primary, secondary, or tertiary), or the type of academic achievement (GPA, grades, and exams) (Robotham, 2008; Madigan and Curran, 2021). Misra and McKean (2000) categorized students’ responses to stress using plans such as emotional (fear, anxiety, worry, guilt, depression), cognitive (coping strategies), behavioral (crying, irritability, abuse of self and others), and psychological (tremors, headaches, weight loss, or weight gain). Studies have shown that these symptoms are associated with poor academic performance (Galbraith and Merrill, 2015). All these responses could affect students’ time management and study skills. Being faced with a variety of stressors such as managing interpersonal relationships, academic work, assignments, and personal development, university students are more likely to experience stress, which later can lead to a decrease in their physical and mental health (Lin and Huang, 2014). In a study conducted on a sample of medical students, the authors found that burnout is associated not only with academic stressors but also with local stressors (Boudreau et al., 2004). Some studies found a direct relationship between students’ outside activities such as employment and academic exhaustion (Jacobs and Dodd, 2003). Higher work involvement has negative effects on student academic performance, being also positively correlated with high levels of stress (Galbraith and Merrill, 2012). Employed students are at a higher risk of burnout because combining employment with the student role diminishes their capacity to efficiently manage time and energy to accomplish both employment and academic tasks (Benner and Curl, 2018).

Inter-role conflict is a major burnout predictor in employed students (Benner and Curl, 2018), the conflict occurring between workplace engagement and academic requirements, leading to high dropout rates (Vickers et al., 2003). The results showed that students who work between 20 and 29 h per week have a 160% higher dropout rate than students who do not work, and this percentage increases significantly compared to the high number of weekly working hours (Lingard, 2007). Jobs with a part-time program or that involve a large number of hours of work cause students to feel tired and even depressed (Rolfe, 2002). In previous studies, Boudreau et al. (2004) identified that burnout is associated with a high number of stressors such as high number of working hours, worries about college grades, and uncertainty about the future, which lead to a low level of satisfaction between personal and professional lives (Lingard, 2007).

### Test Anxiety, Burnout, and Academic Maladjustment

Test anxiety is the most common type of anxiety in the educational setting (Brodersen, 2017; von der Embse et al., 2018). Defined as a state characterized by a feeling of fear and tension, worry, and a perception of threat in exam situations, test anxiety also has negative physiological manifestations (American Psychiatric Association, 2013). Other authors refer to test anxiety as the fear of being negatively evaluated and of failing (Fernández-Castillo and Caurcel, 2015). Test anxiety negatively affects academic performance and is a predictor of increased risk of academic dropout (Esch et al., 2014), studies also relating it to student burnout (Fernández-Castillo and Caurcel, 2015). While earlier studies showed that girls have higher levels of test anxiety than boys (Hembree, 1988; Bandalos et al., 1995), recent studies showed that there are no statistically significant gender differences in terms of test anxiety, the academic performance being similar (Núñez-Peña et al., 2016; Fernández-Castillo, 2021).

Academic anxiety is considered a general feeling of being nervous and worried in the academic context due to external demands such as tests, assignments, and high pressure in obtaining good grades, and every student has a different experience related to test anxiety (Shapiro, 2014). Further studies have shown that excessive academic anxiety levels are related to various outcomes, and these students are more prone to experience burnout than students with low academic anxiety levels (Romano et al., 2020). Low anxiety could have beneficial properties because it helps students to become motivated and thus learn new material effectively, but high levels of anxiety affect the academic achievement of the students (Coon and Mitterer, 2009). Several studies have shown that higher levels of cognitive test anxiety are associated with poor academic performances (Lawal et al., 2017). The difference in performance between a high test-anxious student and a low test-anxious student could be explained based on their locus of control; low test-anxious students are able to focus on test and pay greater attention than high test-anxious students whose attention is divided between their own emotional response and test itself (Ossai, 2011). The stress associated with exams can cause a series of symptoms such as nausea, eating and sleeping patterns changes, stomach pains, and the prospect of taking an exam (Robotham, 2008) or long-term elements related to the exam can enhance the level of stress and test anxiety (Cassady and Johnson, 2002). Although considerable research has focused on student burnout, academic adjustment, and test anxiety, the interactions among these dimensions are not extensively researched. Test anxiety has negative effects on the academic functioning and performance, leading to higher rates of academic dropout (Esch et al., 2014), difficulties in cognitive processes (Fernández-Castillo, 2021), or increased levels of student burnout (Chust-Hernández et al., 2019). Given their negative effects on the academic functioning, academic burnout, and test anxiety could be considered predictors of learning engagement and academic adjustment. Empirical research involving the three components of burnout (emotional exhaustion, cynicism, and low academic self-efficacy) were linked to academic anxiety (Chin et al., 2017): emotional exhaustion and experience of negative effects contribute to test anxiety, while the effects of cynicism on test anxiety were not clear; in contrast, self-efficacy is considered an antecedent of test anxiety. Concerning the associations between test anxiety and academic adjustment, test anxiety, especially the worry component, has a negative impact on academic achievement (Steinmayr et al., 2016). As test anxiety showed negative links with wellbeing (Steinmayr et al., 2016), a negative link between academic adjustment and test anxiety is likely.

### Burnout as a Predictor of Academic Maladjustment

Burnout is associated with psychological maladjustment, including symptoms of depression, anxiety, compulsions, and avoidance (Taris et al., 2004; Shin et al., 2011). Shin et al. (2011) showed that these symptoms are directly associated with cynicism and exhaustion. In role-conflict theory, Lingard (2007) stated that there is a conflict between the roles that the employed student must play. This is manifested by prioritizing one aspect and neglecting the other role; thus, employed students who spend more time working than they spend learning, studying, or completing their university projects suffer in terms of adapting to academic requirements and *vice versa*. Inter-role conflict has become a necessity for students who choose to work during their study years because they have to provide for their daily living needs (Benner and Curl, 2018).

Students who have a job are more exposed to the risk of burnout because they find themselves in the position of combining the requirements of the job with those of the academic environment, not being able to devote all their time and resources to a single goal, that of an employee or a student. Research shows that students who work more than 20 h a week have lower grades and difficulty completing their academic work (Benner and Curl, 2018). Schaufeli and Enzmann (1998) considered that burnout is a conflict that occurs between the different roles that an individual must play and his perception of time pressure. Maslach and Leiter (2017) presented the phenomenon of burnout from a multidimensional perspective, namely, emotional exhaustion, depersonalization, and reduction of personal achievements (Platon and Gorincioi, 2012). Maslach and Leiter (2017) defined emotional exhaustion as a state of emotional energy depletion, accompanied by the impression that one’s own emotional resources are not adequate for managing environmental problems. Depersonalization is manifested in the social sphere and refers to the tendency to treat people as objects, not to be interested in what happens to them and the lack of desire to communicate, accompanied by ignorant behaviors. The reduction of personal achievements is defined as the awareness of the individual of the fact that he cannot cope with problems, that he does not have a good influence on others, and that he does not feel attracted to his work. The individual manifests the feeling of incompetence and loss of hope in his professional perspectives (Platon and Gorincioi, 2012). Students who experience high levels of academic stress report high levels of academic frustration, miss classes, have low academic performance, low levels of motivation, and consider dropping out of college (Schramer et al., 2019).

One of the objectives of this study was to identify whether the level of burnout generated by the academic environment is higher than that generated by the workplace environment, which would contradict the results obtained by Kohler Giancola et al. (2009) who argued that stressors at work are more effective predictors of burnout than stressors generated by the school or personal life.

Burnout is described as physical, emotional, and mental exhaustion caused by long-term involvement in emotionally demanding situations (Maslach and Leiter, 2017). The task of balancing academic and workplace requirements can lead to increased stress and burnout as students find themselves managing their time and frustration can occur (Yang, 2004). The long-term effects of burnout are manifested at the physical level (migraines, change in eating behavior) and at the psycho-behavioral level (decreased ability to remember and concentrate, decreased decision-making power) (Asberg et al., 2010).

Studies on the effects of workplace on students’ academic performance are contradictory. It has been found that students who work full time have more difficulty in terms of academic adjustment (Pascarella et al., 1994). In longitudinal studies, part-time students experience less effects of burnout in the first 3 years of college, but the negative effects are felt if they work for more than 15 h per week (Pascarella et al., 1998). Lundberg (2004) showed that students who worked several hours off campus had difficulty establishing interpersonal relationships with other students or teachers and engaging in academic activities, but no negative and significant effects were found.

Procrastination is a phenomenon that individuals feel, the differences are found in the degrees of manifestation; the level of procrastination that impedes normal functioning is the most severe form that has repercussions in all aspects of an individual’s life (Lakshminarayan et al., 2013). In terms of academic work, this is one of the common problems of students. Most students procrastinate due to high stress levels and low academic performance (Ferrari et al., 1995).

## Methods

### Aims and Hypotheses

The main aim of this study was to analyze the differences between student burnout in different contexts, work- and academic-related burnout. The secondary aim was to examine the predictive role of burnout in academic maladjustment, including also test anxiety as a mediator and occupational status as a moderator.

Hypothesis 1. Employed students have higher levels of academic burnout than work-related burnout, these differences being maintained regardless of the type of work schedule (part-time vs. full-time).

Hypothesis 2. Occupational status moderates the relationship between academic burnout and academic maladjustment.

Hypothesis 3. Test anxiety mediates the relationship between academic burnout and academic maladjustment, occupational status being a moderator.

### Participants and Procedure

The investigated sample consisted of 151 students from different Romanian universities, with 40 (26.49%) male students and 111 (73.51%) female students. A cross-sectional design with a convenience sample was used. The data were collected through on online form posted on several social media pages and groups of the most important Romanian universities. The mean age was 21 years (*SD* = 3.93, *Xmin* = 18, *Xmax* = 51). Our factual data questionnaire also revealed important aspects regarding the participants’ status in the labor market and their work schedule. This study included 63 (41.72%) employed students, out of which 29 (46.03%) had part-time jobs and 34 (53.97%) had full-time jobs. This study also included 88 (58.28%) unemployed students. Concerning the educational level, the sample included 140 (92.7%) undergraduates and 11 (7.3%) graduates (master’s students).

The questionnaires were administered online. Participation in the survey was fully voluntary and not rewarded. The participants gave their written consent that provided information about the aims of the study, guarantees of anonymity, voluntary participation, data treatment, and instructions for filling out the questionnaire.

### Measures

Burnout was measured with the burnout assessment tool (BAT) (Schaufeli et al., 2020) was used to measure burnout at workplace. For a deeper understanding of this topic, we replaced the term “work” with “faculty” to measure the level of burnout for unemployed students. BAT includes two dimensions, namely, core symptoms with 23 items and secondary symptoms with 10 items rated on a 5-point Likert scale (*from 1* = *never to 5* = *always*). The Cronbach’s alpha coefficient for workplace was high for core symptoms (α = 0.94) and secondary symptoms (α = 0.89) and for all subscales, namely, *exhaustion* (α = 0.90) focusing on extreme tiredness, *mental distance* (α = 0.81) focusing on mental withdrawal and psychological detachment from the job, *cognitive impairment* (α = 0.90) focusing on reduced functional capacity to adequately regulate cognitive processes such as memory and attention, *emotional impairment* (α = 0.80) focusing on reduced functional capacity to regulate emotional processes such as anger or sadness, *psychological distress* (α = 0.84) focusing on unpleasant feelings that interferes with daily activities, and *psychosomatic complaints* (α = 0.80) focusing on physical symptoms.

The academic maladjustment was measured with the Academic Adjustment Questionnaire (AAQ) (Clinciu and Cazan, 2014). The AAQ consists of 43 items rated on a 5-point Likert scale (*from 1* = *very little characteristic to me, to 5* = *very much characteristic to me*), covering three dimensions, namely, *academic neuroticism* (14 items, α = 0.91) focusing on anxiety, depression, self-depreciation, irritability, anger, hostility, and vulnerability; *academic procrastination* (10 items, α = 0.87) focusing on tendency to delay or postpone current academic tasks; and *academic dishonesty* (19 items, α = 0.92) focusing on the tendency to cheat and get involved in academic misconduct and dishonest behaviors. Cronbach’s alpha for the entire scale was high (α = 0.94), with high scores indicating academic maladjustment.

Test anxiety was measured with the Cognitive Anxiety Scale (Cassady and Johnson, 2002). It is a unidimensional scale that consists of 27 items rated on a scale (*from 1* = *not at all characteristic to me, to 5* = *totally characteristic to me*). CAT is a 27-item measure focused on the cognitive domain of test anxiety. The items are measured on a 4-point Likert-type scale, with responses ranging from “Not at all like me” to “Very much like me” and focus on behaviors such as engaging in task-irrelevant thinking, comparing self to others, experiencing intruding thoughts, and having relevant cues escape the learner’s attention. The Cronbach’s alpha coefficient obtained in this study was 0.93.

### Data Analysis

We used a cross-sectional design to explore the differences between burnout in different contexts, academic- and work-related context. Repeated-measures ANOVA was used. The normality assumption was checked, showing normal distribution and no sign of multivariate outliers. Mauchly’s test showed that sphericity assumption was met. To test moderation hypothesis of the study, Process 3.0, Model 1, and Model 59 (Hayes, 2017) were utilized. The statistical significance of the index of moderated mediation, and the moderated-mediation and moderated serial-mediation effects, was assessed by interpreting the 95% bias-corrected confidence interval (5,000 samples).

## Findings

Employed students have higher levels of academic burnout than work-related burnout, these differences being maintained regardless of the type of work schedule (part-time vs. full-time).

A repeated-measures ANOVA was used to test hypothesis 1. The differences were significant for all the burnout dimensions, except emotional impairment. Academic burnout was higher for all the dimensions than work-related burnout (Table 1). The type of job (part-time vs. full-time) does not lead to differences between burnout levels in the two contexts, except for cognitive impairment, in this case control being higher in the context of work for part-time students. Therefore, hypothesis 1 is sustained by the data.

**TABLE 1:** Differences between burnout levels in different contexts—Repeated-measures ANOVA.

	Effects	Part-time	Full-time	Work–University		Burnout* status	
			
		M (SD)	M (SD)	*F*_(1,59)_	η*^2^*	*F*_(1,59)_	η*^2^*
Burnout total	Work	2.59 (0.58)	2.36 (0.62)	9.70**	0.14	0.90 ns	0.01
	University	2.86 (0.94)	2.86 (1.08)				
Exhaustion	Work	2.96 (1.05)	2.92 (0.87)	6.28**	0.09	0.19 ns	0.003
	University	3.40 (1.12)	3.23 (1.11)				
Mental distance	Work	2.54 (0.99)	2.08 (0.89)	9.19**	0.14	2.43 ns	0.04
	University	2.77 (1.08)	2.81 (1.31)				
Cognitive impairment	Work	2.48 (1.13)	2.08 (0.89)	17.02***	0.22	5.04*	0.07
	University	2.72 (1.00)	2.90 (1.32)				
Emotional impairment	Work	2.17 (1.04)	2.02 (0.60)	1.40	0.02	0.67	0.01
	University	2.22 (1.01)	2.28 (1.09)				

N_full–time_ = 32, N_part–time_ = 29, *p < 0.05, **p < 0.01, ***p < 0.001.

To analyze the associations between our variables, the Pearson coefficient correlations were computed. The analysis revealed highly significant correlations between academic maladjustment and burnout in both conditions, work and university, but significantly higher for the university-related burnout. Test anxiety also showed positively significant but moderate association both with academic maladjustment and its dimensions, neuroticism being the highest correlated, and with burnout and its dimensions in both conditions, work and university context. Given these associations, we further tested several moderation and mediation models.

**FIGURE 1:**
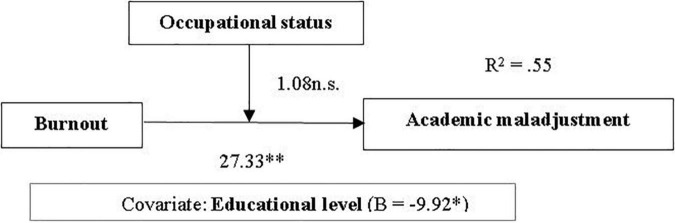
Moderation model of relationship between burnout and academic maladjustment (moderator: occupational status). Note: n.s. = not significant, *p < 0.05, **p < 0.01.

It was tested using Process 3.0 (Model 1) (Figure 1), the occupational status being the moderator for the prediction of academic maladjustment. The educational level was included as a covariable, the undergraduates and graduates differing in terms of their working responsibilities (most of the graduates are employed) and skills to regulate their learning; they have a different background and both work and learning experience. The results showed that there is no moderation effect on the occupational status but burnout has a positive direct significant effect on the prediction of maladjustment (Table 2).

**TABLE 2:** Pearson correlation coefficients between the study variables.

	1	2	3	4	5	6	7	8	9	10	11	12	13	14
Academic (mal)adjustment	1													
Neuroticism	0.77^***^	1												
Procrastination	0.81^***^	0.61^***^	1											
Dishonesty	0.81^***^	0.33^***^	0.49^***^	1										
Burnout total work	0.51*^**^	0.4^***^	0.53^***^	0.36^***^	1									
Exhaustion work	0.44^***^	0.414^**^	0.48*^**^	0.28^*^	0.87^**^	1								
Mental distance work	0.37^**^	0.28^*^	0.35^**^	0.32^*^	0.87^***^	0.68^***^	1							
Cognitive impairment work	0.55^** *^	0.49^***^	0.57^***^	0.38^**^	0.82^***^	0.55^***^	0.63^***^	1						
Emotional impairment work	0.40^**^	0.38^**^	0.40^**^	0.26^*^	0.86^***^	0.64^***^	0.71^***^	0.70^***^	1					
Burnout total Univ.	0.73^***^	0.64^***^	0.70^***^	0.48^***^	0.4^***^	0.33^**^	0.31^*^	0.53^***^	0.43^***^	1				
Exhaustion Univ.	0.63^***^	0.61^***^	0.60^***^	0.37^***^	0.428^***^	0.38^**^	0.25	0.46^***^	0.36^**^	0.89^***^	1			
Mental distance Univ.	0.66^***^	0.45^***^	0.62^***^	0.53^***^	0.42^***^	0.31^*^	0.34^**^	0.45^***^	0.36^**^	0.87^***^	0.69^***^	1		
Cognitive impairment Univ.	0.65^***^	0.52^***^	0.69^***^	0.42^***^	0.44^***^	0.28^*^	0.33^**^	0.56^***^	0.40^***^	0.86^***^	0.64^***^	0.77^***^	1	
Emotional impairment Univ.	0.60^***^	0.60^***^	0.52^***^	0.37^***^	0.33^**^	0.15	0.21	0.43^***^	0.44^***^	0.80^***^	0.62^***^	0.60^***^	0.62^***^	1
Test anxiety	0.64^***^	0.72^***^	0.52^***^	0.33^***^	0.43^***^	0.38^**^	0.25^*^	0.48^***^	0.33^**^	0.58^***^	0.57^***^	0.36^***^	0.48^***^	0.55^***^

*p < 0.05, **p < 0.001, ***p < 0.0001; the number of participants for the work-related burnout is 61, while for the other variables it is 149.

**FIGURE 2:**
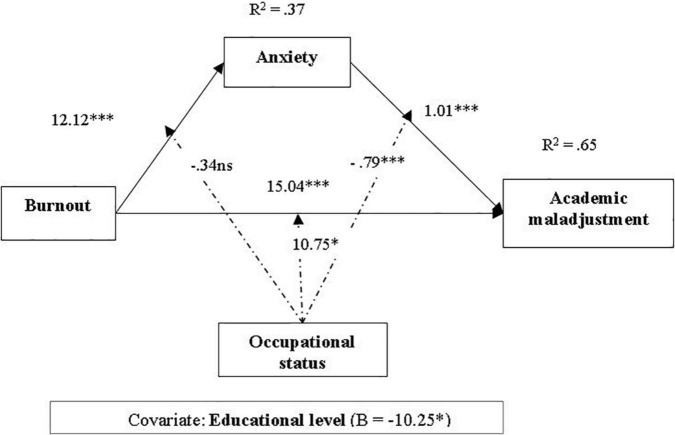
Moderated mediation model of relationship between burnout and academic maladjustment (mediator: test anxiety; moderator: occupational status). n.s. = not significant, *p < 0.05, **p < 0.01, ***p < 0.001.

To test hypothesis 3 (test anxiety mediates the relationship between academic burnout and academic maladjustment, occupational status being a moderator) (Figure 2), we used Process 3.0, Model 59 (Table 3). The results of the moderated mediation analysis showed that academic burnout significantly predicts test anxiety, students with higher burnout showing higher levels of test anxiety; academic burnout significantly predicts maladjustment, students with higher levels of burnout being less adjusted; test anxiety mediates the relationship between burnout and adjustment, high burnout explains higher levels of anxiety, which in turns explains higher academic maladjustment; and the occupational status moderates the relationship between anxiety and burnout, the prediction being stronger for non-employed students. Similarly, the occupational status moderates the relationship between burnout and adjustment, the prediction being stronger for non-employed students; in addition, the mediating role of anxiety in the relationship between burnout and adjustment is stronger for employed students, hypothesis 3 being sustained (Table 4).

**TABLE 3:** Path estimates and explained variance from the moderation analysis.

Model variables	Path	LLCI	ULCI	*R* ^2^	*F (df)*	*p*
Model 1 Predicting academic maladjustment				0.55	45.02 (4,144)	<0.001
Burnout	27.33	21.94	32.72			<0.001
Occupational status	−4.54	−29.80	20.71			0.722
Burnout × Status	1.08	−7.46	9.62			0.803
Covariable: Educational level	−9.92	−19.23	−0.61			0.036

*LLCI, lower and level for confidence interval; ULCI, upper level for confidence interval.*

**TABLE 4:** Path estimates and explained variance from the moderated mediation analyses.

Model variables	Path	LLCI	ULCI	*R* ^2^	*F (df)*	*p*
Predicting test anxiety				0.37	21.16 (4,144)	<0.001
Burnout	12.12	8.64	15.61			<0.001
Status	6.69	−9.61	23.01			0.418
Burnout × Status	−0.34	−5.86	5.17			0.901
Covariable: Educational level	−0.31	−6.37	5.65			0.906
Predicting academic maladjustment				0.65	45.71 (4,144)	<0.001
Burnout	15.04	8.96	21.1			<0.001
Anxiety	1.01	0.70	1.32			<0.001
Status	19.60	−7.89	47.09			0.161
Burnout × Status	10.75	1.51	20.00			0.023
Anxiety × Status	−0.79	−1.24	−0.34			0.001
Covariable: Educational level	−10.25	−0.18.47	−2.02			0.015
Index of moderated mediation *a_3_b* = −9.75, bias corrected bootstrap *CI* = [−17.62,						
−3.06]						

*LLCI, lower and level for confidence interval; ULCI, upper level for confidence interval.*

## Discussion

### Key Findings of the Study

This research has contributed to the understanding of the academic maladjustment of employed and non-employed students. The main objective of this study was to analyze the burnout differences in different categories of students. The results showed that academic burnout is higher than work-related burnout. High levels of test anxiety explain high levels of academic burnout, which in turn explains low levels of academic adjustment. The results highlight the mediating role of anxiety in the relationship between academic burnout and academic maladjustment with occupational status as a moderator.

The higher level of academic burnout was also revealed by previous studies (Schramer et al., 2019). Kohler Giancola et al. (2009) argued that work stressors are stronger predictors of burnout than stressors generated by the school or personal life. Other studies showed that factors such as anxiety, self-esteem, and time management skills are involved in the adjustment process and the achievement of high academic performance of employed students (Jacobs and Dodd, 2003; Boudreau et al., 2004). For hypothesis 1, an interesting aspect was found; cognitive impairment is higher for students who work part-time. A similar result was found in the study of Rolfe (2002), which means that students with part-time jobs face more difficulties than students with full-time jobs or unemployed students, the effects of part-time working at universities being poor attendance, late arrival, early leaving, and late submissions of work.

In the case of hypothesis 2, the results showed that the occupational status of the student, employed or unemployed, has no statistically significant moderating effect on the relationship between academic burnout and academic maladjustment, only the direct effect of academic burnout on maladaptation being significant. This result contradicts previous studies (Schaufeli and Enzmann, 1998; Lingard, 2007), arguing that employed students are at a much higher risk of burnout because they are in a position to balance academic tasks with job requirements. These results may suggest that employed students have the ability to manage their time much better than unemployed students, and the direct effect of burnout can only be explained by the high academic demands. Comparing the results with those obtained for adult workers, temporary employees are more stressed due to the more aggravating job characteristics and less quality work than permanent employees (De Witte and Näswall, 2003), leading to less job control and more psychological distress; therefore, employed students (such as permanent employees) could have developed better time management skills and more efficient coping strategies due to the need to be more organized.

In the case of hypothesis 3, the results showed that high levels of test anxiety explain high levels of academic burnout, which in turn explains low levels of academic adjustment. The paths were statistically significant only in the case of unemployed students, which could be explained by the fact that they are more likely to have poor academic performance and procrastinate more, due to the high level of stress and worry, as other studies have shown (Ferrari et al., 1995). The results were similar to those of other studies, test anxiety being considered an important predictor of student burnout (Chust-Hernández et al., 2019) and academic achievement (Cassady and Johnson, 2002).

### Limitations and Suggestions for Future Studies

This study has several limitations. First, the unequal sample in terms of gender distribution and also the relatively small sample size. Our sample consists of a higher number of female participants which may influence the results obtained in previous studies (Cassady and Johnson, 2002), showing that female students have higher levels of emotionality while male students typically score lower on measurements of test anxiety and burnout. The assessment of academic burnout also needs further investigations. The BAT was previously used in academic context (Romano et al., 2020); however, the need for investigating the psychometric properties of the Romanian version used for students in academic settings remains a limitation of this study and a future research direction. Although burnout is an occupational phenomenon and is mainly applied to working contexts, research in the field showed that the academic context is comparable to the working context and students are similar to professionals in terms of burnout risk (Schaufeli et al., 2002). The moderating role of the occupational status needs further investigations. Comparing our results with those obtained for adult workers, temporary employees are more stressed due to the more aggravating job characteristics and less quality work than permanent employees (De Witte and Näswall, 2003), leading to less job control and more distress; therefore, employed students (such as permanent employees) could have developed better time management skills and more efficient coping strategies due to the need to be more organized.

Another limitation to consider and a direction for future studies represent the type of employment, part-time or full-time, and the domain of the job because the level of stress may differ depending on the field in which they operate on the labor market. Thus, students who work in a field similar to their academic specialization exhibit lower levels of stress and implicit burnout (Boudreau et al., 2004). The level of burnout felt by employed students is not only associated with stressors in the academic environment but also with the workplace stressors. Therefore, future studies should analyze the relationship between burnout and the type of job, revealed as important factors also in previous studies. Moreover, since we did not use a balanced sample, future studies should focus on gathering an equivalent sample or emphasize the gender differences regarding burnout symptoms and test anxiety. Finally, subsequent research should verify the relationship between burnout and personality traits, which can also explain the manifestation of burnout symptoms, as previous studies have shown (Zellars et al., 2000; Teven, 2007).

### Implications

This study contributes to the understanding of the phenomenon of burnout among employed students and the consequences of occupational status on academic performance, adjustment, and completion rates in university.

The results of this study are congruent with the results of previous studies on this topic. Students with full-time jobs have higher levels of academic burnout than work burnout for all dimensions, except cognitive impairment, which is higher for students with part-time jobs. These results are relevant and support Lingard’s (2007) theory of inter-role conflict, which argues that employed students find themselves in a position to meet the demands of both the faculty and the workplace. Academic burnout in this situation is an effective and direct predictor of academic maladaptation, a result supported by the study of Benner and Curl (2018), and causes a dramatic decrease in motivation to learn and a considerable increase in school dropout (Schramer et al., 2019). The results of this study showed that this relationship between burnout and academic maladaptation is mediated by anxiety, which means that students with a high level of anxiety face more difficulties to adjust to the academic environment, which leads to a significant increase in the stress that can lead to burnout.

This study was carried out on a sample of students from Romania who had the role of bringing into discussion a critical issue in the educational system, namely, the increasing rate of academic dropout. The increasing tuition fees and expensive living needs force the students to find a job in order to support themselves. To reduce the stress caused by the interference between work and academic demands, it is necessary to propose intervention programs and strategies to help employed students cope with the multiple challenges they face. These strategies could include didactic changes such as the use of educational podcasts, recorded courses for a higher accessibility for students, offering them the possibility to structure the learning materials using efficient learning strategy, and offering tasks to compensate for academic activities.

## Data Availability Statement

The raw data supporting the conclusions of this article will be made available by the authors, without undue reservation.

## Ethics Statement

The studies involving human participants were reviewed and approved by the Council of the Faculty of Psychology and Education Sciences. The patients/participants provided their written informed consent to participate in this study.

## Author Contributions

G-LD developed the study concept, conducted the data collection, and drafted the manuscript. A-MC performed the data analysis. Both authors took part in result interpretation, reviewed and edited several versions of the manuscript and provided critical revisions, and approved the final version of the manuscript for submission.

## Conflict of Interest

The authors declare that the research was conducted in the absence of any commercial or financial relationships that could be construed as a potential conflict of interest.

## Publisher’s Note

All claims expressed in this article are solely those of the authors and do not necessarily represent those of their affiliated organizations, or those of the publisher, the editors and the reviewers. Any product that may be evaluated in this article, or claim that may be made by its manufacturer, is not guaranteed or endorsed by the publisher.

## References

[B1] American Psychiatric Association (2013). *Diagnostic and Statistical Manual of Mental Disorders (DSM-5§).* Washington, DC: American Psychiatric Association.

[B2] Asberg M. Garpe T. Krakau I. Nygren A. Rohnde M. Wahlberg A. (2010). Stress as the cause of mental illness. *Lakartidningen* 107 1307–1310. 20556983

[B3] Bandalos D. L. Yates K. Thorndike-Christ T. (1995). Effects of math self-concept, perceived self-efficacy, and attributions for failure and success on test anxiety. *J. Educ. Psychol.* 87 611–623. 10.1037/0022-0663.87.4.611

[B4] Benner K. Curl A. L. (2018). Exhausted, stressed, and disengaged: does employment create burnout for social work students? *J. Soc. Work Educ.* 54 300–309. 10.1080/10437797.2017.1341858

[B5] Boudreau D. Santen S. A. Hemphill R. R. Dobson J. (2004). Burnout in medical students: examining the prevalence and predisposing factors during the four years of medical school. *Ann. Emerg. Med.* 44 S75–S76. 10.1016/j.annemergmed.2004.07.248

[B6] Brodersen L. D. (2017). Interventions for test anxiety in undergraduate nursing students: an integrative review. *Nurs. Educ. Perspect.* 38 131–137. 10.1097/01.NEP.0000000000000142 36785470

[B7] Brougham R. R. Zail C. M. Mendoza C. M. Miller J. R. (2009). Stress, sex differences, and coping strategies among college students. *Curr. Psychol.* 28 85–97. 10.1007/s12144-009-9047-0

[B8] Cassady J. C. Johnson R. E. (2002). Cognitive test anxiety and academic performance. *Contemp. Educ. Psychol.* 27 270–295. 10.1006/ceps.2001.1094

[B9] Chin E. C. H. Williams M. W. Taylor J. E. Harvey S. T. (2017). The influence of negative affect on test anxiety and academic performance: an examination of the tripartite model of emotions. *Learn. Individ. Dif.* 54 1–8. 10.1016/j.lindif.2017.01.002

[B10] Chust-Hernández P. Castellano-Rioja E. Fernández-García D. Chust-Torrent J. I. (2019). Ansiedad ante los exámenes en estudiantes de Enfermería: factores de riesgo emocionales y de sueño. *Ansiedad Estrés* 25 125–131. 10.1016/j.anyes.2019.05.001

[B11] Clinciu A. I. (2013). Adaptation and stress for the first year university students. *Procedia Soc. Behav. Sci.* 78 718–722. 10.1016/j.sbspro.2013.04.382

[B12] Clinciu A. I. Cazan A.-M. (2014). Academic adjustment questionnaire for the university students. *Procedia Soc. Behav. Sci.* 127 655–660. 10.1016/j.sbspro.2014.03.330

[B13] Coon D. Mitterer J. (2009). Psychology of test anxiety. *J. Cengage Learn.* 28 48–53.

[B14] De Witte H. Näswall K. (2003). ‘Objective’ vs ‘subjective’ job insecurity: consequences of temporary work for job satisfaction and organizational commitment in four European countries. *Econ. Ind. Democracy* 24 149–188. 10.1177/0143831X03024002002

[B15] Dyrbye L. N. Thomas M. R. Massie F. S. Power D. V. Eacker A. Harper W. (2008). Burnout and suicidal ideation among U.S. medical students. *Ann. Intern. Med.* 149 334–341. 10.7326/0003-4819-149-5-200809020-00008 18765703

[B16] Esch P. Bocquet V. Pull C. Couffignal S. Lehnert T. Graas M. (2014). The downward spiral of mental disorders and educational attainment: a systematic review on early school leaving. *BMC Psychiatry* 14:237. 10.1186/s12888-014-0237-4 25159271 PMC4244046

[B17] Fernández-Castillo A. (2021). State-anxiety and academic burnout regarding university access selective examinations in spain during and after the COVID-19 lockdown. *Front. Psychol.* 12:621863. 10.3389/fpsyg.2021.621863 33584481 PMC7873299

[B18] Fernández-Castillo A. Caurcel M. J. (2015). State test-anxiety, selective attention and concentration in university students: state test-anxiety and attention. *Int. J. Psychol.* 50 265–271. 10.1002/ijop.12092 25104475

[B19] Ferrari J. R. Johnson J. L. McCown W. G. (1995). *Procrastination and Task Avoidance: Theory, Research, and Treatment.* New York, NY: Plenum Press.

[B20] Fiorilli C. De Stasio S. Di Chiacchio C. Pepe A. Salmela-Aro K. (2017). School burnout, depressive symptoms and engagement: their combined effect on student achievement. *Int. J. Educ. Res.* 84 1–12. 10.1016/j.ijer.2017.04.001

[B21] Galbraith C. S. Merrill G. B. (2012). Academic and work-related burnout: a longitudinal study of working undergraduate university business students. *J. Coll. Stud. Dev.* 53 453–463. 10.1353/csd.2012.0044 34409987

[B22] Galbraith C. S. Merrill G. B. (2015). Academic performance and burnout: an efficient frontier analysis of resource use efficiency among employed university students. *J. Further High. Educ.* 39 255–277. 10.1080/0309877X.2013.858673

[B23] Gorter R. C. Albrecht G. Hoogstraten J. Eijkman M. A. J. (1999). Factorial validity of the maslach burnout inventory-dutch version (MBI-NL) among dentists. *J. Organ. Behav.* 20 209–217. 10.1002/(sici)1099-1379(199903)20:2<209::aid-job984>3.0.co;2-y

[B24] Hayes A. F. (2017). *Introduction to Mediation, Moderation, and Conditional Process Analysis: A Regression–Based Approach*, 2nd Edn. New York, NY: Guilford Publications.

[B25] Hembree R. (1988). Correlates, causes, effects, and treatment of test anxiety. *Rev. Educ. Res.* 58 47–77. 10.3102/00346543058001047

[B26] Hicks T. Heastie S. (2008). High school to college transition: a profile of the stressors, physical and psychological health issues that affect the first-year on-campus college student. *J. Cult. Divers.* 15 143–147. 19025202

[B27] Jacobs S. R. Dodd D. (2003). Student burnout as a function of personality, social support, and workload. *J. Coll. Stud. Dev.* 44 291–303. 10.1353/csd.2003.0028 34409987

[B28] Kohler Giancola J. Grawitch M. J. Borchert D. (2009). Dealing with the stress of college: a model for adult students. *Adult Educ. Q.* 59 246–263. 10.1177/0741713609331479

[B29] Lakshminarayan N. Potdar S. Reddy S. G. (2013). Relationship between procrastination and academic performance among a group of undergraduate dental students in India. *J. Dent. Educ.* 77 524–528. 10.1002/j.0022-0337.2013.77.4.tb05499.x 23576599

[B30] Larcombe W. Finch S. Sore R. Murray C. M. Kentish S. Mulder R. A. (2016). Prevalence and socio-demographic correlates of psychological distress among students at an Australian university. *Stud. High. Educ.* 41 1074–1091. 10.1080/03075079.2014.966072

[B31] Lawal A. M. Idemudia E. S. Adewale O. P. (2017). Academic self-confidence effects on test anxiety among Nigerian university students. *J. Psychol. Africa* 27 507–510. 10.1080/14330237.2017.1375203

[B32] Lin S.-H. Huang Y.-C. (2014). Life stress and academic burnout. *Act. Learn. High. Educ.* 15 77–90. 10.1177/1469787413514651

[B33] Lingard H. (2007). Conflict between paid work and study: does it impact upon students’ burnout and satisfaction with university life? *J. Educ. Built Environ.* 2 90–109. 10.11120/jebe.2007.02010090

[B34] Lundberg C. A. (2004). Working and learning: the role of involvement for employed students. *NASPA J.* 41 201–215. 10.2202/1949-6605.1330

[B35] Madigan D. J. Curran T. (2021). Does burnout affect academic achievement? a meta-analysis of over 100,000 students. *Educ. Psychol. Rev.* 33 387–405. 10.1007/s10648-020-09533-1

[B36] Maslach C. Leiter M. P. (2017). “Understanding burnout: new models,” in *The Handbook of Stress and Health*, eds Cooper C. L. Quick J. C. (Chichester: John Wiley & Sons, Ltd), 36–56. 10.1002/9781118993811.ch3

[B37] Misra R. McKean M. (2000). College students’ academic stress and its relation to their anxiety, time management, and leisure satisfaction. *Am. J. Health Stud.* 16 41–54.

[B38] Murberg T. A. Bru E. (2004). School-related stress and psychosomatic symptoms among norwegian adolescents. *Sch. Psychol. Int.* 25 317–332. 10.1177/0143034304046904

[B39] Neumann Y. Finaly-Neumann E. Reichel A. (1990). Determinants and consequences of students’ burnout in universities. *J. High. Educ.* 61:20. 10.2307/1982032

[B40] Njim T. Mbanga C. M. Tindong M. Fonkou S. Makebe H. Toukam L. (2019). Burnout as a correlate of depression among medical students in Cameroon: a cross-sectional study. *BMJ Open* 9:e027709. 10.1136/bmjopen-2018-027709 31061054 PMC6502056

[B41] Núñez-Peña M. I. Suárez-Pellicioni M. Bono R. (2016). Gender differences in test anxiety and their impact on higher education students’ academic achievement. *Procedia Soc. Behav. Sci.* 228 154–160. 10.1016/j.sbspro.2016.07.023

[B42] Ossai M. C. (2011). Guidance and counselling implications of examination anxiety as a predictor of students’ attitude towards examination malpractices. *Mediterr. J. Soc. Sci.* 2 85–90.

[B43] Oswalt S. Riddock C. (2007). What to do about being overwhelmed: graduate students, stress and university services. *Coll. Stud. Affiars J.* 61 20–31. 10.47678/cjhe.v44i3.186036

[B44] Pascarella E. T. Bohr L. Nora A. Desler M. Zusman B. (1994). Impacts of on campus and off campus work on first year cognitive outcomes. *J. Coll. Stud. Dev.* 35 364–370.

[B45] Pascarella E. T. Edison M. I. Nora A. Hagedorn L. S. Terenzini P. T. (1998). Does work inhibit cognitive development during college? *Educ. Eval. Policy Anal.* 20 75–93. 10.3102/01623737020002075

[B46] Platon C. Gorincioi V. (2012). Stresul şi sindromul arderii emoţionale la profesorii universitari. *Rev. Stiint. a Univ. de Stat din Moldova*. 59, 224–228.

[B47] Robotham D. (2008). Stress among higher education students: towards a research agenda. *High. Educ.* 56 735–746. 10.1007/s10734-008-9137-1

[B48] Rolfe H. (2002). Students’ demands and expectations in an age of reduced financial support: the perspectives of lecturers in four english universities. *J. High. Educ. Policy Manag.* 24 171–182. 10.1080/1360080022000013491

[B49] Romano L. Tang X. Hietajärvi L. Salmela-Aro K. Fiorilli C. (2020). Students’ trait emotional intelligence and perceived teacher emotional support in preventing burnout: the moderating role of academic anxiety. *Int. J. Environ. Res. Public Health* 17:4771. 10.3390/ijerph17134771 32630744 PMC7369914

[B50] Salanova M. Schaufeli W. Martínez I. Bresó E. (2010). How obstacles and facilitators predict academic performance: the mediating role of study burnout and engagement. *Anxiety Stress Coping* 23 53–70. 10.1080/10615800802609965 19326271

[B51] Schaufeli W. B. Enzmann D. (1998). *The Burnout Companion to Study and Practice: A Critical Analysis.* Boca Raton, FL: CRC press.

[B52] Schaufeli W. B. Taris T. W. (2005). The conceptualization and measurement of burnout: common ground and worlds apart The views expressed in *Work & Stress* Commentaries are those of the author(s), and do not necessarily represent those of any other person or organization, or of the journal. *Work Stress* 19 256–262. 10.1080/02678370500385913

[B53] Schaufeli W. B. Desart S. De Witte H. (2020). Burnout assessment tool (BAT)—development, validity, and reliability. *Int. J. Environ. Res. Public Health* 17:9495. 10.3390/ijerph17249495 33352940 PMC7766078

[B54] Schaufeli W. B. Martínez I. M. Pinto A. M. Salanova M. Bakker A. B. (2002). Burnout and engagement in university students: a cross-national study. *J. Cross Cult. Psychol.* 33 464–481. 10.1177/0022022102033005003

[B55] Schramer K. M. Rauti C. M. Kartolo A. B. Kwantes C. T. (2019). Examining burnout in employed university students. *J. Public Ment. Health* 19 17–25. 10.1108/JPMH-05-2019-0058

[B56] Shapiro A. L. (2014). Test anxiety among nursing students: a systematic review. *Teach. Learn. Nurs.* 9 193–202. 10.1016/j.teln.2014.06.001

[B57] Shin H. Kim B. Lee M. Noh H. Kim K. Lee S. M. (2011). A short-term longitudinal study of mental health and academic burnout among middle school students. *Korean J. Sch. Psychol.* 8 133–152. 10.1186/s13054-016-1208-6 27885969 PMC5493079

[B58] Steinmayr R. Crede J. McElvany N. Wirthwein L. (2016). Subjective well-being, test anxiety, academic achievement: testing for reciprocal effects. *Front. Psychol.* 6:1994. 10.3389/fpsyg.2015.01994 26779096 PMC4705295

[B59] Taris T. W. Horn J. E. V. Schaufeli W. B. Schreurs P. J. G. (2004). Inequity, burnout and psychological withdrawal among teachers: a dynamic exchange model. *Anxiety Stress Coping* 17 103–122. 10.1080/1061580031000151620

[B60] Teven J. J. (2007). Teacher temperament: correlates with teacher caring, burnout, and organizational outcomes. *Commun. Educ.* 56 382–400. 10.1080/03634520701361912

[B61] van Rooij E. C. M. Jansen E. P. W. A. van de Grift W. J. C. M. (2018). First-year university students’ academic success: the importance of academic adjustment. *Eur. J. Psychol. Educ.* 33 749–767. 10.1007/s10212-017-0347-8

[B62] Vickers M. Lamb S. Hinkley J. (2003). *Student Workers in High School and Beyond?: The Effects of Parttime Employment on Participation in Education, Training and Work. Longitudinal Surveys of Australian Youth Research Report No. 30.* Melbourne, VIC: Australian Council for Educational Research.

[B63] von der Embse N. Jester D. Roy D. Post J. (2018). Test anxiety effects, predictors, and correlates: a 30-year meta-analytic review. *J. Affect. Disord.* 227 483–493. 10.1016/j.jad.2017.11.048 29156362

[B64] Yang H.-J. (2004). Factors affecting student burnout and academic achievement in multiple enrollment programs in Taiwan’s technical–vocational colleges. *Int. J. Educ. Dev.* 24 283–301. 10.1016/j.ijedudev.2003.12.001

[B65] Youssef C. M. Luthans F. (2007). Positive organizational behavior in the workplace: the impact of hope, optimism, and resilience. *J. Manag.* 33 774–800. 10.1177/0149206307305562

[B66] Zellars K. L. Perrewe P. L. Hochwarter W. A. (2000). Burnout in health care: the role of the five factors of personality. *J. Appl. Soc. Pyschol.* 30 1570–1598. 10.1111/j.1559-1816.2000.tb02456.x

